# Supplementary material for: Early Dynamics of Quantitative SEPT9 and SHOX2 Methylation in Circulating Cell-Free Plasma DNA during Prostate Biopsy for Prostate Cancer Diagnosis
Source: Cancers (Basel). 2022 Sep 7;14(18):4355. doi: 10.3390/cancers14184355 (PMC9496792; doi:10.3390/cancers14184355)
Supplement: Supplementary file 1 [file cancers-14-04355-s001.zip › cancers-1854434-supplementary.pdf]

cancers

MDPI

*Article*

# Early dynamics of quantitative *SEPT9* and *SHOX2* methylation in circulating cell-free plasma DNA during prostate biopsy for prostate cancer diagnosis

## Figure S1. Trial scheme

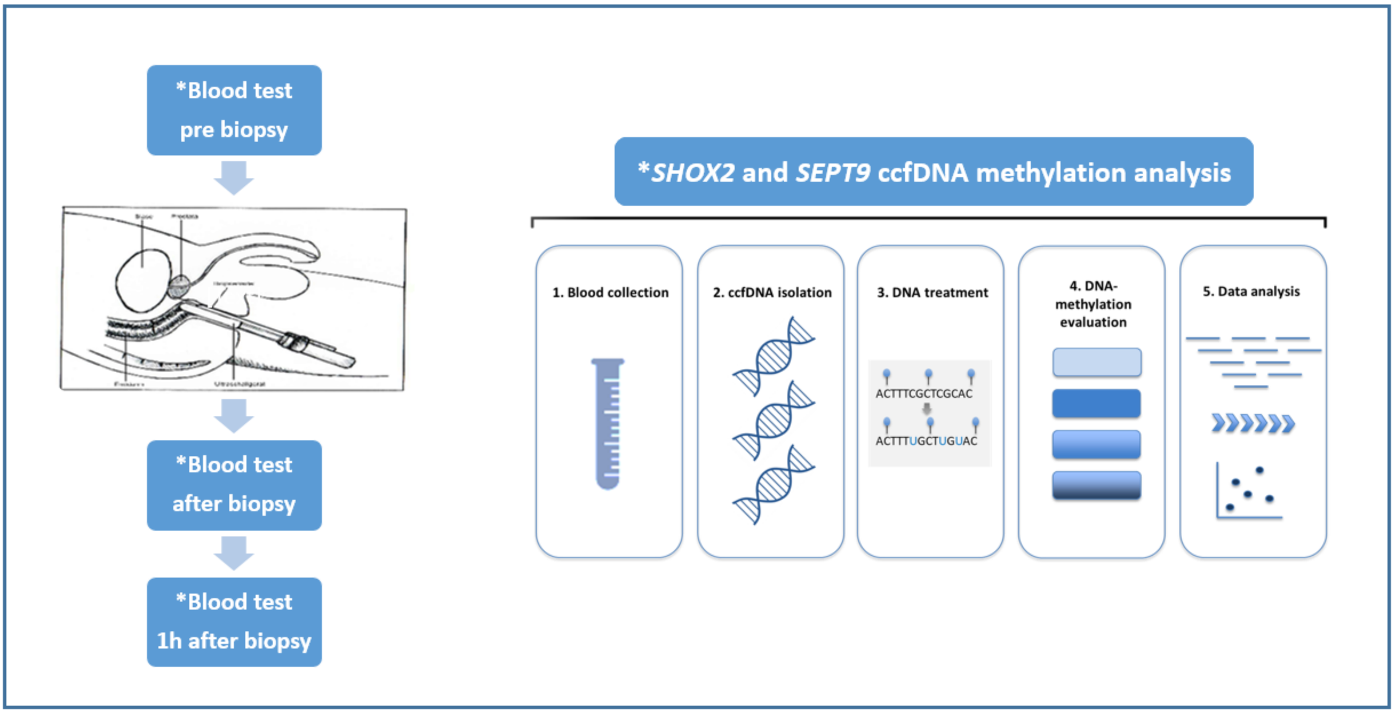


**Figure S1** represents the study design. Blood samples were collected before, immediately after, and one hour after prostate biopsy. Each of these blood samples (*) were quantitatively analyzed for *SHOX2* and *SEPT9* methylation in ccfDNA.

ccfDNA, circulating cell-free DNA; *SHOX2, short stature homeobox 2*; *SEPT9, Septin 9*

**Figure S2. Quantitative methylated *SHOX2* in PCa tissue samples depending on tumor burden and grading**

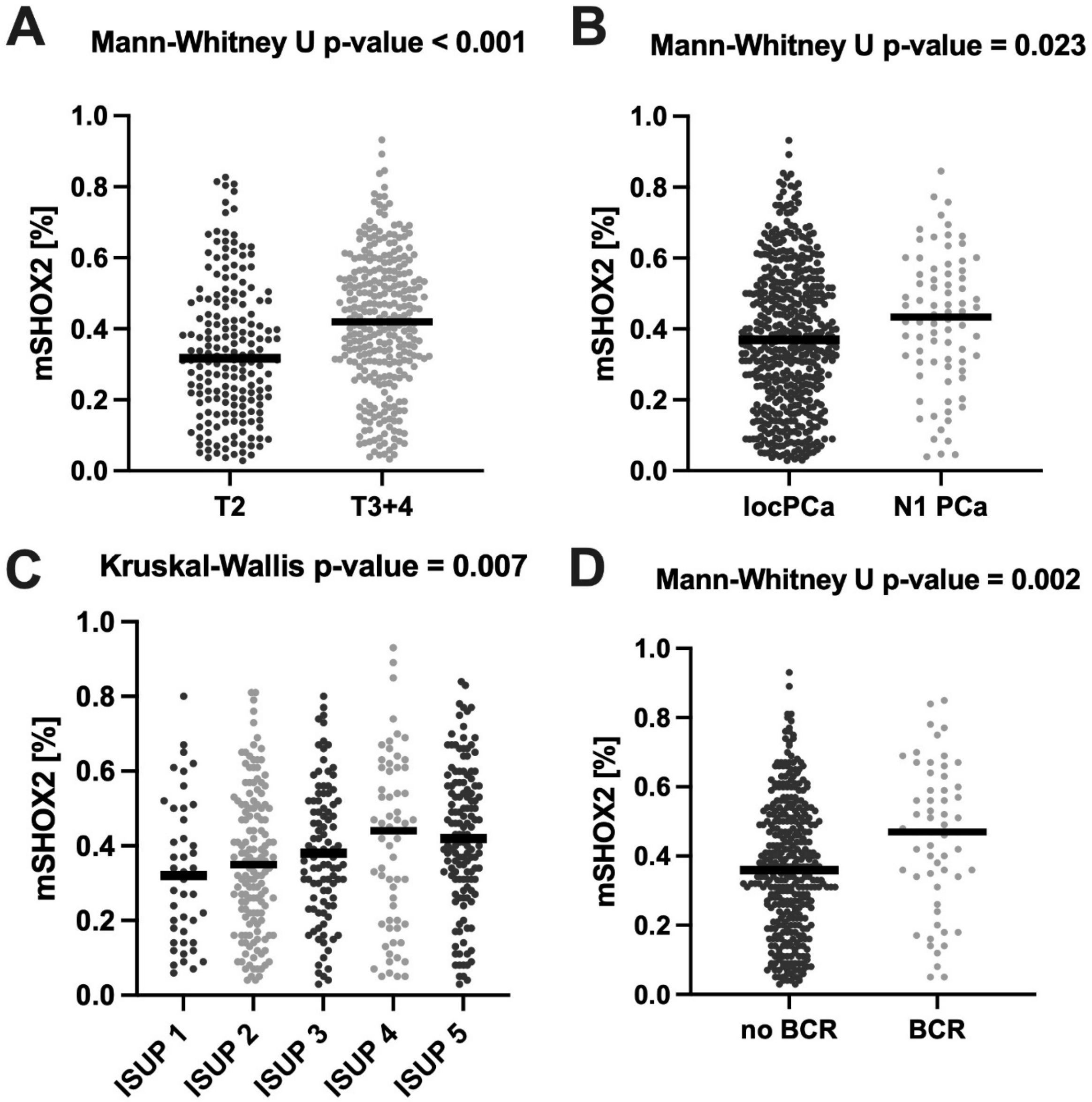


**Figure S2** illustrates scatterplots showing quantitative differences of *mSHOX2 (methylated short stature homeobox 2)* in tissue samples between localized (T2) and locally advanced prostate cancer (T3/4) (**A**); between localized (loc) prostate cancer (PCa) and patients with lymph node metastasis (N1, **B**); with regards to ISUP (International Society of Urological Pathology) grading groups (**C**); and patients with and without biochemical recurrence (BCR) of the PCa (**D**).

**Figure S3.** ***SHOX2*** **mRNA expression in PCa tissue samples depending on tumor burden and grading**

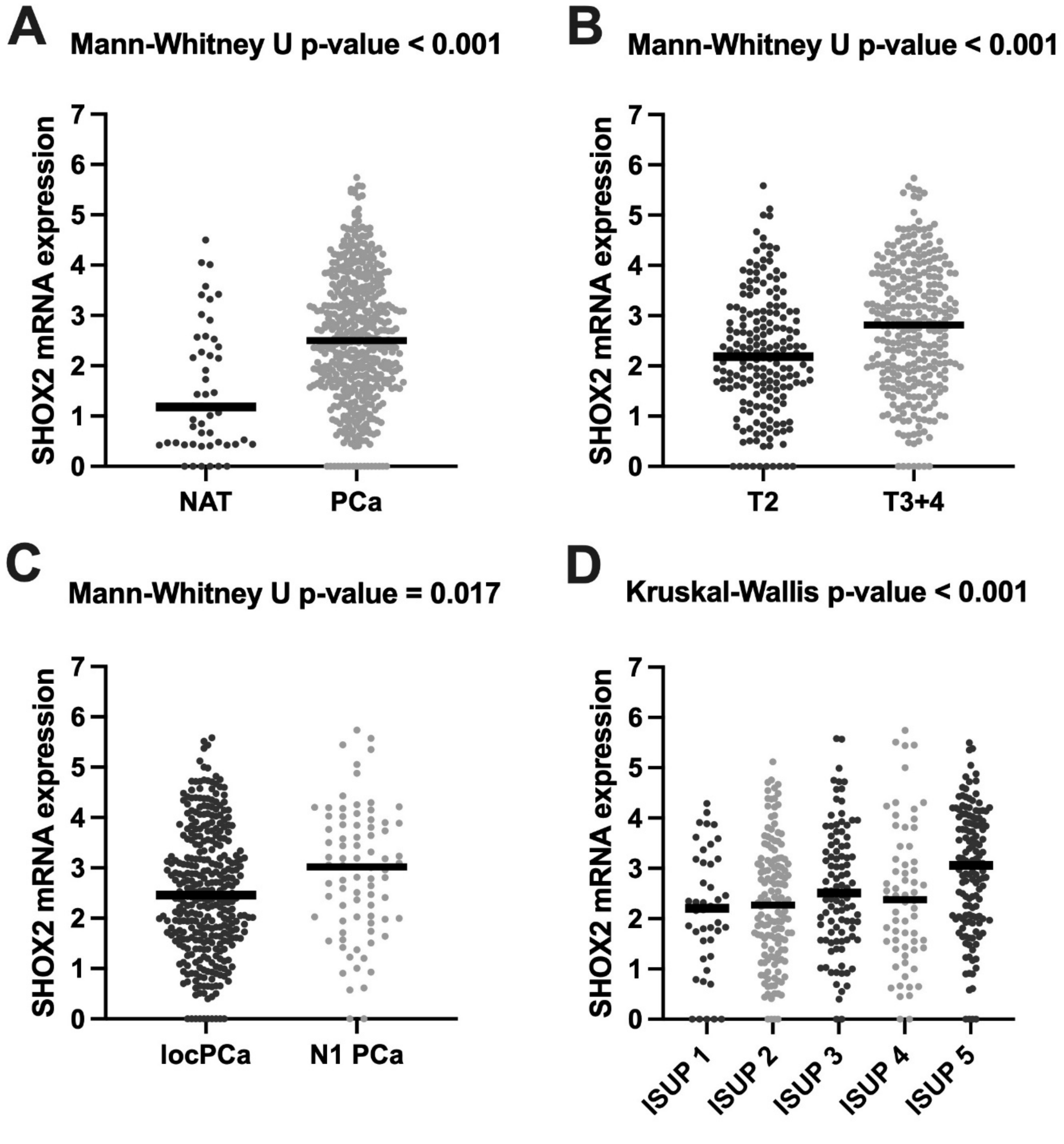


**Figure S3** illustrates scatterplots showing quantitative differences of *SHOX2 (short stature homeobox 2)* mRNA expression in tissue samples between NAT (normal adjacent tissue) and primary PCa (prostate cancer, **A**); localized (T2) and locally advanced prostate cancer (T3/4, **B**); between localized (loc) prostate cancer (PCa) and patients with lymph node metastasis (N1, **C**); and with regards to ISUP (International Society of Urological Pathology) grading groups, (**D**).

**Table S1. Intercorrelations (by Pearson) between *mSEPT9* and *mSHOX2* ccfDNA level during prostate biopsy and metric clinical PCa surrogate markers**

| | PSA | PSAD | MRI lesion size | Tumor-bearing cores |
|---|---|---|---|---|
| *mSEPT9* ccfDNA [%] at T1 | **.328*** | **.403*** | .293 | .199 |
| *mSEPT9* ccfDNA [%] at T2 | **.328*** | **.403**** | .300 | .208 |
| *mSEPT9* ccfDNA [%] at T3 | **.331*** | **.413**** | **.396*** | **.310*** |
| *mSHOX2* ccfDNA [%] at T1 | **.322*** | **.397*** | .302 | .205 |
| *mSHOX2* ccfDNA [%] at T2 | **.322*** | **.398*** | .317 | .214 |
| *mSHOX2* ccfDNA [%] at T3 | **.312*** | **.390*** | **.396*** | **.313*** |

*p < 0.05, ** p < 0.01. |R| = .10 small, |R| = .30 medium, |R| = .50 large correlation.
*mSEPT9* ccfDNA, methylated *Septin 9* circulating cell-free DNA; *mSHOX2* ccfDNA, methylated *short stature homeobox 2* circulating cell-free DNA; PSA, prostate specific antigen; PSAD, PSA density; MRI, multiparametric magnetic resonance imaging of the prostate.
T1: blood collection before prostate biopsy, T2: blood collection directly after prostate biopsy, T3: blood collection one hour after prostate biopsy.

**Table S2. Intercorrelations (by Spearman) between *mSEPT9* and *mSHOX2* ccfDNA level during prostate biopsy and ranked clinical PCa surrogate markers and ISUP grading**

| | Abnormal DRE | Abnormal US | Highest PI-RADS | Highest ISUP |
|---|---|---|---|---|
| *mSEPT9* ccfDNA positivity score at T1 | .202 | **.432*** | **.331*** | .222 |
| *mSEPT9* ccfDNA positivity score at T2 | **.316*** | **.324*** | **.385**** | **.357*** |
| *mSEPT9* ccfDNA positivity score at T3 | **.462**** | **.362*** | **.376*** | **.332*** |
| *mSHOX2* ccfDNA positivity score at T1 | -.041 | -.088 | .228 | .085 |
| *mSHOX2* ccfDNA positivity score at T2 | -.049 | -.052 | .141 | .104 |
| *mSHOX2* ccfDNA positivity score at T3 | **.355*** | **.419**** | **.432**** | .028 |

*$p < 0.05$, ** $p < 0.01$. $|R| = .10$ small, $|R| = .30$ medium, $|R| = .50$ large correlation.
*mSEPT9* ccfDNA, methylated *Septin 9* circulating cell-free DNA; *mSHOX2* ccfDNA, methylated *short stature homeobox 2* circulating cell-free DNA; DRE, digital rectal examination; US, transrectal ultrasound; ISUP, International Society of Urological Pathology
T1: blood collection before prostate biopsy, T2: blood collection directly after prostate biopsy, T3: blood collection 1h after prostate biopsy.
